# A meta-analysis of effects of 3-nitrooxypropanol on methane production, yield, and intensity in dairy cattle

**DOI:** 10.3168/jds.2022-22211

**Published:** 2022-12-07

**Authors:** Ermias Kebreab, André Bannink, Eleanor May Pressman, Nicola Walker, Alexios Karagiannis, Sanne van Gastelen, Jan Dijkstra

**Affiliations:** 1 Department of Animal Science, University of California, Davis 95616; 2 Wageningen Livestock Research, Wageningen University & Research, PO Box 338, 6700 AH, Wageningen, the Netherlands; 3 DSM Nutritional Products, Animal Nutrition & Health, PO Box 2676, 4002 Basel, Switzerland; 4 Animal Nutrition Group, Wageningen University & Research, PO Box 338, 6700 AH, Wageningen, the Netherlands

**Keywords:** 3-nitrooxypropanol, dairy, meta-analysis, methane

## Abstract

Ruminants, particularly dairy and beef cattle, contribute to climate change through mostly enteric methane emissions. Several mitigating options have been proposed, including the feed additive 3-nitrooxypropanol (3-NOP). The objectives of this study were to explain the variability in the mitigating effect of 3-NOP and to investigate the interaction between diet composition and 3-NOP dose, using meta-analytical approaches. Data from 13 articles (14 experiments) met the selection criteria for inclusion in the meta-analysis, and 48 treatment means were used for the analysis. Mean differences were calculated as 3-NOP treatment mean minus control treatment mean and then expressed as a percentage of the control mean. Three types of models were developed: (1) one including 3-NOP dose, overall mean, and individual covariate; (2) a combination of neutral detergent fiber (NDF), 3-NOP dose, and overall mean; and (3) one selected model from all combinations of up to 5 covariates, which were compared using a leave-one-out cross validation method. Models including only 3-NOP dose resulted in a significant reduction of 32.7%, 30.9%, and 32.6% for CH_4_ production (g/d), yield (g/kg dry matter intake), and intensity (g/kg energy-corrected milk), respectively, at an average 3-NOP dose of 70.5 mg/kg dry matter (DM). The greater the NDF content in the diet, the lower the reduction efficiency for a given 3-NOP dose. For 10 g/kg DM increase in NDF content from its mean (329 g of NDF/kg of DM) the 3-NOP effect on CH_4_ production was impaired by 0.633%, the 3-NOP effect on CH_4_ yield by 0.647%, and the 3-NOP effect on CH_4_ intensity by 0.723%. The analysis based on leave-one-out cross validation showed an increase in NDF and crude fat content reduces efficacy of 3-NOP and an increase in 3-NOP dose increases efficacy. A 1% (10 g/kg) DM decrease in dietary NDF content from its mean may increase the efficacy of 3-NOP in reducing CH_4_ production by 0.915%. A 1% (10 g/kg DM) decrease in dietary crude fat content from its mean enhances the efficacy of 3-NOP on CH_4_ production by 3.080% at a given dose and NDF level. For CH_4_ yield, next to 3-NOP dose, dietary NDF content and dietary crude fat content were included in the selected model, but also dietary starch content with an opposite direction to NDF and crude fat. The effect of 3-NOP dose on CH_4_ intensity was similar to its effect on CH_4_ production, whereas the effect of dietary NDF content was slightly lower. Expanding the previously published models with the newly available data published from trials since then improved model performance, hence demonstrating the value of regularly updating meta-analyses if a wider range of data becomes available.

## INTRODUCTION

The dairy industry has been scrutinized for its environmental impact. Several studies have indicated that the livestock sector contributes to environmental change (e.g., de Vries and de Boer, 2010), including greenhouse gas **(GHG**) emissions. Agriculturally derived methane (CH_4_) emissions mostly result from enteric fermentation and, to a lesser extent, storage of manure from ruminant livestock. Methane from enteric fermentation accounts for 44% of the total GHG emissions from livestock (GLEAM, 2022). Livestock supply chains are estimated to account for 14.5% (based on 100-year Global Warming Potential) of total human-induced GHG emissions (Gerber et al., 2013). The world ruminant population increased by 66% from 1960 to 2017 and is projected to continue to increase to meet global demand for meat and milk, which will further exacerbate GHG emissions from animal agriculture (FAOSTAT, 2017).

Due to its considerable contribution, enteric CH_4_ production has been targeted to reduce GHG emissions from the dairy sector. Several CH_4_ mitigation strategies have been proposed, including changes to animal breeding and management, reformulation of diets, improvement of forage quality, and rumen manipulation (Hristov et al., 2013a,b). Recently, Honan et al. (2021) and Arndt et al. (2022) reviewed strategies used to mitigate CH_4_, including feed additives that are given in small quantities to change rumen metabolism to inhibit methanogenesis. One such compound, 3-nitrooxypropanol **(3-NOP**), has been reported to reduce enteric CH_4_ emissions by over 30% on average (Dijkstra et al., 2018) in dairy cattle based on 5 studies. The small molecule 3-NOP has a molecular shape similar to that of methyl-coenzyme M, which is a substrate of coenzyme M reductase (**MCR**), the enzyme involved in the last step of methanogenesis (Duin et al., 2016). Molecular docking studies suggest that as an analog of methyl-coenzyme M, 3-NOP selectively binds to the active site of MCR in a position that places its reducible nitrate group in electron transfer distance to Ni(I) and inactivates MCR by oxidizing the active site nickel +1 in co-factor F_430_. Additionally, the nitrate group of 3-NOP is reduced to nitrite in the process, and in this form further inactivates MCR (Duin et al., 2016). However, it is known that methanogens contain a repair system that can reactivate MCR in a H_2_−, ATP-, and chaperone-dependent reduction process (Prakash et al., 2014). Therefore, once 3-NOP has been completely metabolized and is not present anymore, CH_4_ emissions return to their original level (Kuhner et al., 1993; Zhou et al., 2013). As a result of MCR inactivation by 3-NOP, H_2_ accumulates, shifting the flows of metabolic H_2_ in rumen fermentation from acetate toward propionate, butyrate, or valerate (Romero-Perez et al., 2014; Schilde et al., 2021), resulting in lower H_2_ production as substrate for methanogens. Although H_2_ may accumulate with 3-NOP, only a small fraction of the produced H_2_ becomes emitted by the animal (van Gastelen et al., 2020).

Several studies have attempted to quantify the effects of 3-NOP on CH_4_ emission in cattle (e.g., Dijkstra et al., 2018; Jayanegara et al., 2018), but their results were still based on a relatively small number of experiments. Dijkstra et al. (2018) had to combine beef and dairy cattle data because there were not enough experiments available on dairy only at that time. Even with a limited number of experiments, Dijkstra et al. (2018) proposed that 3-NOP dose rate and diet composition could affect the level of CH_4_ mitigation achievable. A recent study by van Gastelen et al. (2022) in lactating cows confirmed that dose and diet composition are important factors to consider. In their study, animals were fed 3 distinct mixed diets at 2 different 3-NOP dose rates and the authors found marked differences in the achieved level of CH_4_ mitigation. Thus, variation in the reduction of CH_4_ emissions in response to the addition of 3-NOP compared with a control diet necessitates using a greater number of studies to explain the variability in the mitigating effect of 3-NOP. This need, coupled with an increased number of studies conducted in dairy cattle fed 3-NOP with different diets, allowed further investigation of the interaction between diet composition and 3-NOP dose. We hypothesized that supplementing 3-NOP reduces enteric CH_4_ production (g/d), yield (g/kg DM), and intensity (g/kg ECM) but that the level of CH_4_ reduction depends on 3-NOP dose and the nutrient profile of the diet to which 3-NOP is supplemented.

## MATERIALS AND METHODS

This meta-analysis used only published data, so institutional animal care and use guidelines are not applicable.

### Data Sources

Literature searches of the Web of Science (Thomson Reuters Science, https://www.webofscience.com/), Scopus (Elsevier, https://www.scopus.com/), and Google Scholar (https://google.com) online databases were conducted using keywords “3NOP” (including all variants, such as “nitrooxypropanol” and the brand name Bovaer) + “dairy cattle.” After rejecting publications that reported in vitro experiments only, were literature reviews, performed life cycle assessments only, or reported CH_4_ emissions from manure only, this literature search resulted in 25 publications related to the effect of 3-NOP on CH_4_ emissions in dairy cattle. For inclusion in the database, the studies were required to include a control treatment group that did not receive 3-NOP, and a treatment group with 3-NOP top-dressed or mixed in the diet, and to include measured CH_4_ production. Six publications were rejected because they did not report CH_4_ production of dairy cattle. Five publications, mostly MS or PhD theses or abstracts, were rejected because these repeated data from other publications included in our analysis. One article was rejected because of 2 reasons: first, 3-NOP was delivered directly into the rumen and not via the feed as in other studies, and second, 3-NOP was dosed twice daily, resulting in a pulse dosing effect rather than a continuous feeding effect as in other studies. One 3-NOP treatment group from a study was removed because, unlike in the other 3-NOP treatment group and all other studies, 3-NOP was included only in concentrate feed delivered via out-of-parlor concentrate feeding stations, and not in the basal diet. This resulted in a pulse dosing effect when animals visited the concentrate feeding station, rather than the continuous feeding effect that was achieved in all of the other studies when applied in the basal TMR. Data from 13 articles (14 experiments) met the selection criteria, and 48 treatment means were used for dairy cattle (Haisan et al., 2014, 2017; Hristov et al., 2015; Lopes et al., 2016; Van Wesemael et al., 2019; Melgar et al., 2020a,b, 2021; van Gastelen et al., 2020, 2022; Yanibada et al., 2020; Bampidis et al., 2021; Schilde et al., 2021). Methane emissions were estimated using the GreenFeed technique (10 experiments; C-Lock Inc.), the climate-controlled respiration chamber technique (2 experiments), or the sulfur hexafluoride tracer gas technique (2 experiments). Methane intensity was reported in g/kg of milk, g/kg ECM, g/kg of 4% FCM, or g/kg fat- and protein-corrected milk (CVB, 2018). All intensities were converted to g/kg ECM by using the estimates of the reported intensity components or were provided by the researchers who authored the publication. The analyzed dose of 3-NOP (mg/kg DM) was the one provided by in-feed analytics if reported in respective publications. Otherwise, the target dose was used.

Several studies did not include information on overall dietary starch, crude fat, or OM. Any missing nutrient composition values of experimental diets were calculated using the ingredient composition and nutritive value tables in NASEM (2021) for starch, crude fat, and ash composition. Starch, crude fat, and ash composition of feeds not included in NASEM (2021) were obtained from Feedipedia (https://www.feedipedia.org) or the manufacturer’s website for commercial concentrates. Total dietary starch, crude fat, and OM were calculated by weighing the contribution of the respective starch, crude fat, and OM compositions of each ingredient by the proportion of the ingredient in total dietary DM. Calculated values were compared with analyzed values reported in the studies. On average, calculated and analyzed crude fat, starch, and OM contents differed by −7.1%, −1.2%, and 0.06%, respectively.

None of the selected studies provided overall values of dietary rumen fermentable OM or total-tract OM digestibility, but these are potentially important determinants of rumen fermentation and digestion, and hence of enteric CH_4_ production, yield, and intensity. Therefore, values were obtained from Dutch Centraal Veevoederbureau feeding tables (CVB, 2018). A small number of ingredients in diets from studies included in this analysis were not included in these feeding tables. The fraction of dietary components with known values was on average 96.6% on a DM basis. Total dietary rumen fermentable OM and total-tract OM digestibility (% OM) of the diets were calculated by weighing the contribution of each ingredient with known fermentable OM and digestible OM (calculated from OM content and OM fermentability and digestibility of each of the ingredients) by the proportion of each ingredient in dietary DM. The dietary OM digestibility (% OM) was calculated by dividing total dietary digestible OM by total dietary OM. For consistency, OM values for this portion of the analysis were also taken from the CVB (2018) tables. Total dietary fermentable OM was calculated by first multiplying the proportion of each ingredient within the diet by the fermentable OM of each ingredient [calculated using the CVB (2018) system]. Subsequently this was divided by the proportion of the diet with known values to account for missing values by assuming this could be scaled to a diet with 100% known values. A summary of the database is presented in Table 1.

### Model Development and Selection

**Figure 1.**
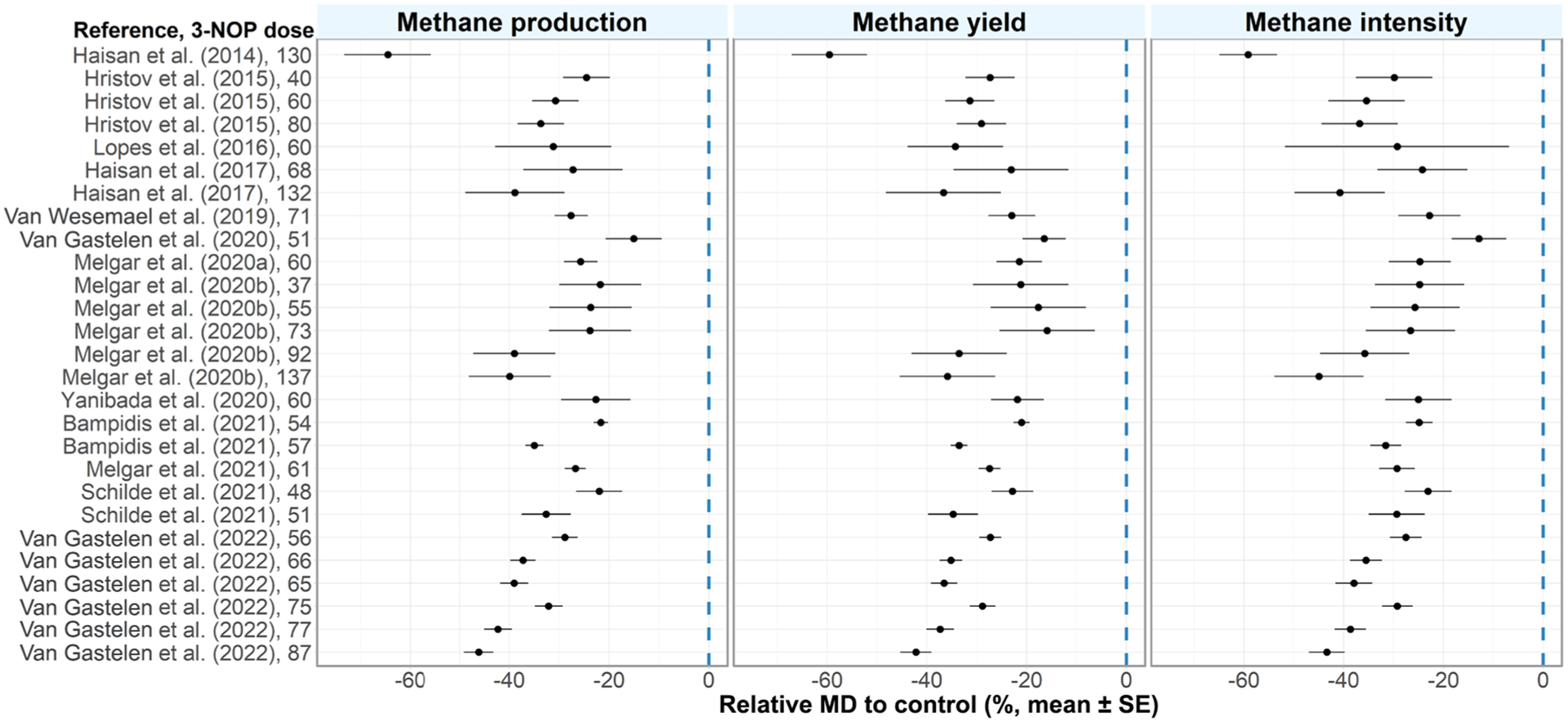
Relative mean differences to control in CH_4_ production (g/d), yield (g/kg DMI), and intensity (g/kg ECM). Dose of 3-nitrooxypropanol (3-NOP) is depicted in milligrams per kilogram DM. MD = mean difference.

The data were analyzed in a similar manner to Dijkstra et al. (2018). In brief, mean difference (**MD**) data were derived as 3-NOP treatment mean minus control treatment mean. Further, MD were divided by control means, which resulted in relative MD expressed as a percentage of the control mean. This was done for CH_4_ production, yield, and intensity (Figure 1). Relative MD were meta-analyzed using weights inversely proportional to the variance reported for each study. Analyses were executed in R (version 4.1.1, R Foundation for Statistical Computing) using the package “metafor” and a robust variance estimation was used to account for multiple treatment groups sharing a common control group (Viechtbauer, 2010). During model fitting, one data point (high-dose group of Melgar et al., 2020b) was deemed an outlier based on Cook’s distance and removed from all analyses. The considered covariates were 3-NOP dose (mg/kg DM), mean DMI (kg/d), NDF (% of DM), crude fat (% of DM), CP (% of DM), starch (% of DM), OM (% of DM), fermentable OM (% of DM), total-tract OM digestibility (% OM), and roughage proportion (% of DM). Covariates that are correlated (absolute Pearson correlation coefficient greater than 0.5) were not used in the same model (see Supplemental Figure S1, https://data.mendeley.com/datasets/zjy2hs3642). Effects of each individual nutritional covariate from models including the respective covariate and the effect of 3-NOP dose are presented. The amount of residual heterogeneity (**τ**^**2**^) and its proportion (***I***^**2**^) to unaccounted variability were calculated using the “metafor” package. Models with all combinations of covariates (Barton, 2020), including up to 5 covariates, were compared using a leave-one-out cross validation (**LOOCV**). The results (covariate coefficients, *P*-values, and LOOCV residuals) from models with the lowest LOOCV-based root mean square error (**RMSE**) of the relative MD (%), for effects with *P* < 0.10 of covariates and with less than 0.50 absolute correlation with other covariates, are presented. Also, a quadratic effect for 3-NOP dose was explored (results not shown) but did not improve model performance. For illustration purposes, the residuals and RMSE from the models developed by Dijkstra et al. (2018) for CH_4_ production and yield as well as updated versions of the models from the present study are presented.

## RESULTS AND DISCUSSION

The meta-analysis in the current study combined findings from several experiments to describe the effect of 3-NOP in dairy cattle related to CH_4_ production, yield, and intensity. The relative MD in CH_4_ production, yield, and intensity were all negative, indicating that 3-NOP had a consistent antimethanogenic effect (Figure 1). Models including only 3-NOP dose resulted in a reduction of 32.7% (*P* < 0.001), 30.9% (*P* < 0.001), and 32.6% (*P* < 0.001) for CH_4_ production, yield, and intensity, respectively, at an average 3-NOP dose of 70.5 mg/kg DM. The reduction in CH_4_ production was similar to the value reported in the previous meta-analysis by Dijkstra et al. (2018) despite their average dose being 15% greater. For CH_4_ yield, the relative value was 5% greater in efficacy than previously established. The effect of 3-NOP dose in all individual models was significant (results not presented) and showed that the larger the 3-NOP dose, the greater the relative reduction of each unit of expression of CH_4_.

The effects of 3-NOP were associated with large heterogeneity. Accounting for no covariates resulted in more than 70% of the total variability of the 3-NOP effects in CH_4_ production, yield, and intensity being due to heterogeneity, indicating that there may be variables that can explain this heterogeneity. Several explanatory variables were evaluated, which were first combined individually with 3-NOP dose and then combined with all possible combinations of all explanatory variables as far as they were not correlated (absolute Pearson correlation coefficient less than 0.50). Effects of explanatory variables, from models that included each nutritional component individually as well as 3-NOP dose and from models with the lowest LOOCV RMSE, on relative MD of CH_4_ production, yield, and intensity are provided in Tables 2, 3, and 4, respectively. In models for relative MD in CH_4_ production, which also included 3-NOP dose, DMI (*P* = 0.398), dietary CP content (*P* = 0.217), dietary crude fat content (*P* = 0.240), dietary OM content (*P* = 0.601), dietary fermentable OM content (*P* = 0.227), OM digestibility (*P* = 0.371), roughage proportion (*P* = 0.965), and dietary starch content (*P* = 0.728) were not significant when added individually (Table 2). Dietary NDF content was significant (*P* = 0.023), where both τ^2^ and LOOCV RMSE reduced from 23% and 7.95%, respectively, when only 3-NOP dose was included to 21% and 7.34%, respectively, when both 3-NOP dose and NDF content were included in the model (*I*^2^ = 61%).

For CH_4_ yield, DMI (*P* = 0.377), dietary content of CP (*P* = 0.502), crude fat (*P* = 0.378), OM (*P* = 0.731), fermentable OM (*P* = 0.364), starch (*P* = 0.328), roughage proportion (*P* = 0.568), and OM digestibility (*P* = 0.542) were not significant when added individually with 3-NOP dose (Table 3). Dietary NDF content was significant (*P* = 0.003), where both τ^2^ and LOOCV RMSE reduced from 22% and 8.32%, respectively, when only 3-NOP dose was included to 19% and 7.76%, respectively, when both 3-NOP dose and NDF content were included in the model (*I*^2^ = 60%). Also for CH_4_ intensity, dietary NDF content was significant (*P* < 0.001), where both τ^2^ and LOOCV RMSE reduced from 8.2% and 6.69%, respectively, when only 3-NOP dose was included to 5.9% and 5.94%, respectively, when both 3-NOP dose and NDF content were included in the model (*I*^2^ = 20%). An additional explanatory variable, DMI, had a significant effect (*P* = 0.037) when added individually with 3-NOP dose, suggesting that greater DMI leads to more reduction in CH_4_ intensity (Table 4). For DMI, τ^2^ and LOOCV RMSE were equal to 4.8% and 6.80%, respectively, when both 3-NOP dose and DMI were included in the model (*I*^2^ = 17%), but DMI did not contribute in the selected (lowest LOOCV RMSE) model.

For all 3 units of CH_4_ emission (production, yield, and intensity), dietary NDF content contributed significantly in addition to 3-NOP dose, as depicted in the results of individual models (Tables 2–4). For CH_4_ production and yield, we updated the Dijkstra et al. (2018) models that were based on 3-NOP dose and NDF content. Similar to Dijkstra et al. (2018), we found that the greater the NDF content in the diet, the lower the reduction efficiency for a given 3-NOP dose. However, in comparison, the updated model results had a lesser effect of dietary NDF content. Dijkstra et al. (2018) reported that a greater dietary NDF content impaired the 3-NOP effect on CH_4_ production by 1.64% and the 3-NOP effect on CH_4_ yield by 1.52% per 10 g/kg DM increase in NDF content from its mean (331 g of NDF/kg of DM). In the present study, however, a greater dietary NDF content impaired the 3-NOP effect on CH_4_ production by 0.633% and the 3-NOP effect on CH_4_ yield by 0.647% per 10 g/kg DM increase in NDF content from its mean (329 g of NDF/kg of DM). This difference is probably because the current study uses an expanded database taking a wider range of NDF values into account (Table 1) and focuses on dairy studies only.

For CH_4_ production, the lowest LOOCV RMSE model included dietary NDF and crude fat content with the same directional effect on the efficacy of 3-NOP to reduce CH_4_ production (Table 2). For CH_4_ production, τ^2^ further reduced to 17 with an *I*^2^ of 55% and a LOOCV RMSE to 6.94%. The directions of the effects of NDF and crude fat suggest that the more NDF or crude fat in a diet, the less effective 3-NOP will be at a given dose in reducing enteric CH_4_ production. After adjusting for dietary NDF and crude fat content, the coefficient of 3-NOP dose for CH_4_ production was 0.282, which translates to an additional 2.82% reduction in CH_4_ production for an increase of 10 mg/kg 3-NOP dose from its mean. The effect of 3-NOP dose was about 10% greater than previously reported (Dijkstra et al., 2018). A 1% (10 g/kg) decrease in dietary NDF content (on DM basis) from its mean may increase the efficacy of 3-NOP in reducing CH_4_ production by 0.915%. It has been previously hypothesized that NDF levels influence the rate at which CH_4_ is reduced with the inclusion of inhibitors due to differences in concentration of methyl-coenzyme M in the rumen (Vyas et al., 2018). Another factor that may be considered is the state of H_2_ dynamics that vary with dietary content of NDF or a complementary nutrient such as starch (van Gastelen et al., 2022). The results suggest that a 1% (10 g/kg) DM decrease in dietary crude fat content from its mean enhances the efficacy of 3-NOP on CH_4_ production by 3.080% at a given dose and NDF level. The relatively greater impact of crude fat content on efficacy of 3-NOP compared with NDF level may be related to the profound effect of crude fat on rumen methanogenesis (Arndt et al., 2022). However, in the current database, the range of crude fat was small (approximately 3%). In addition, missing nutrient components were estimated with tabular values and fat source was not accounted for in the models. Therefore, the current assumptions of effects are efforts to explain the presented data-driven findings. Similarly, the models are applicable for the ranges of the various components in the database—for example, 3-NOP dose approximately 40 to 130 mg/kg DM, NDF approximately 26.5% to 43.5% DM, and crude fat approximately 3% to 6%. Extrapolating outside these ranges should be done with caution, as it is likely that additional unknown relations are existent and the source or form of nutrients may play a role.

For CH_4_ yield, next to 3-NOP dose, dietary NDF content and dietary crude fat content were included in the selected model, but also dietary starch content with an opposite direction to NDF and crude fat (Table 3). Vyas et al. (2018) suggested that adding 3-NOP to high-starch diets might inhibit MCR with greater efficacy because of a lower concentration of MCR. The selected model for CH_4_ intensity included 3-NOP dose and dietary NDF content, as no other model with more covariates (fulfilling the selection criteria) had a better performance (Table 4). In the selected models, the effect of 3-NOP dose on CH_4_ intensity was similar to its effect on CH_4_ production, whereas the effect of dietary NDF content was slightly lower.

The antimethanogenic properties of 3-NOP and dietary variables that moderate its effect can be expressed in the following equations based on Tables 2–4:

$$\begin{array}{l} {\text{ Change (\%) in CH}}_{4}\text{ production }\\ =-32.4-0.282\times (3-\text{NOP}-70.5)\\ +0.915\times (\text{NDF}-32.9)+3.080\times (\text{ crude fat }-4.2), \end{array}$$

where 3-NOP = 3-nitroxypropanol dose (mg/kg of DM), and NDF and crude fat are in % DM.

$$\begin{array}{l} {\text{ Change (\%) in CH}}_{4}\text{ yield }=\\ -30.8-0.226\times (3-\text{NOP}-70.5)\\ +0.906\times (\text{NDF}-32.9)+3.871\times (\text{ crude fat }-4.2)\\ -0.337\times (\text{starch}-21.1), \end{array}$$

where 3-NOP = 3-nitroxypropanol dose (mg/kg of DM), and NDF, crude fat, and starch are in % DM.

$$\begin{array}{l} {\text{ Change (\%) in CH}}_{4}\text{ intensity }=-33.0\\ -0.275\times (3-\text{NOP}-70.5)+0.723\times (\text{NDF}-32.9), \end{array}$$

where 3-NOP = 3-nitroxypropanol dose (mg/kg of DM), and NDF is in % DM.

**Figure 2.**
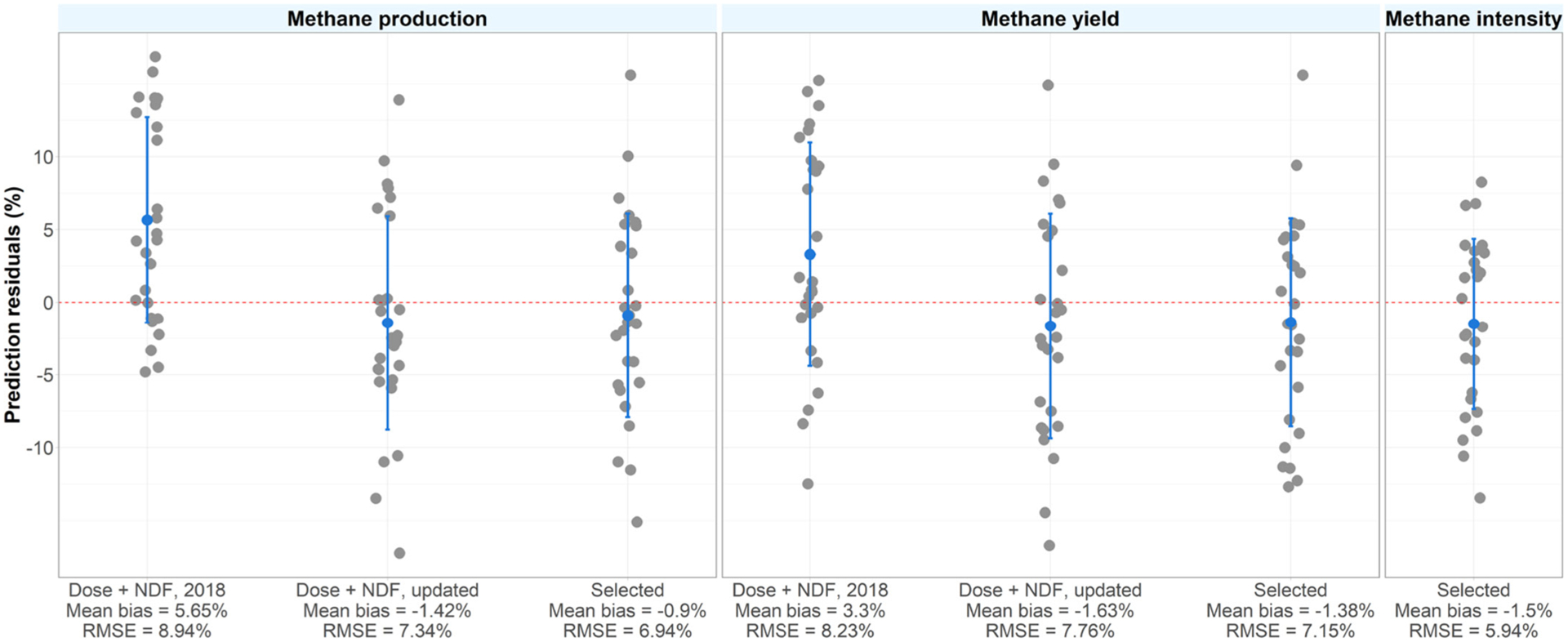
Prediction residuals (mean ± SD) from a leave-one-out cross validation in the models developed for methane production, yield, and intensity and residuals for the models developed by Dijkstra et al. (2018). RMSE = root mean square error.

Supplemental Table S1 (https://data.mendeley.com/datasets/zjy2hs3642) illustrates the expected relative MD of CH_4_ production in various combinations of dietary NDF and crude fat content and for 60 and 80 mg 3-NOP/kg DM dose as an example. The LOOCV prediction residuals of the selected models for each outcome are presented in Figure 2. For CH_4_ production and yield, the residuals of 3 models were compared: (1) based on the equations of Dijkstra et al. (2018) for dairy cattle with 3-NOP and NDF as explanatory variables, (2) the same explanatory variables as (1) as derived in the current analysis, and (3) the model selected based on LOOCV prediction and RMSE. The previously published models underestimated the observed effect of 3-NOP in reducing CH_4_ production and yield. For the current data set, using the Dijkstra et al. (2018) model the mean bias was 5.7% (RMSE = 8.94%) and 3.3% (RMSE = 8.23%) for CH_4_ production and yield, respectively. The selected models from the current study had smaller biases of −0.9% (LOOCV RMSE = 6.94%) and −1.4% (LOOCV RMSE = 7.15%) in predicting CH_4_ production and yield, respectively. Expanding the previously published models with the newly available data published from trials since then improved model performance, hence demonstrating the value of updating meta-analyses if a wider range of data becomes available. It also demonstrates the importance of providing dietary composition details alongside reduction effect sizes to be able to expand databases for building or updating meta-analytical models. Data from trials covering a wide range of dietary starch, NDF, or crude fat levels in the diet enhance the universal usability of the models. This is confirmed by a recent trial of van Gastelen et al. (2022), which specifically aimed to evaluate the effect of diet composition on the reduction effect size of 3-NOP.

The equations provided in the current study can be used to calculate CH_4_ emission reduction for 3-NOP-supplemented dairy cows and incorporated in protocols used for purposes such as carbon market or farm GHG accounting tools. Nevertheless, it is possible to build more complex models to attempt to better represent the underlying mechanisms and explain the findings of the present study. Although this adds complexity, it would allow for more detailed analysis of variation in 3-NOP efficacy considering aspects of rumen microbial metabolism (Duin et al., 2016) and rumen function with particular emphasis on metabolic pathways that yield CH_4_, H_2_, and different types of VFA (van Lingen et al., 2019). The present study followed a strictly empirical approach and is restricted to the experimental observations made, and on usability of the model, given the limited data set. No presumptions were made about the underlying mechanisms and influencing factors that may be involved. The results indicate, however, that moving toward mechanistic modeling should be a goal. Future efforts of meta-analysis with more data could also focus on incorporating other effects typically represented in dynamic mechanistic models of rumen fermentation.

## CONCLUSIONS

The current meta-analysis indicates that the overall effectiveness of 3-NOP at mitigating CH_4_ emissions was 32.7%, 30.9%, and 32.6% for CH_4_ production, yield, and intensity, respectively, at an average 3-NOP dose of 70.5 mg/kg DM. However, the mitigating effect of 3-NOP was modified by the nutrient composition of the diet. Increases in NDF and crude fat concentrations above the average in the database reduced effectiveness of 3-NOP at mitigating methane production and yield, whereas increases in starch content enhanced 3-NOP effectiveness in mitigating methane yield. For methane intensity, reducing the NDF content of the diet enhanced effectiveness of 3-NOP. As expected, for all units of methane emission, increasing the dose of 3-NOP resulted in larger efficacy.

## Figures and Tables

**Table 1.** Descriptive statistics of feed intake, dietary characteristics, and CH_4_ emission^1^

Item	Mean	Median	SD	Minimum	Maximum
DMI (kg/d)	22.8	23.1	2.9	18.2	28.0
CP (% of DM)	17.0	16.5	1.7	13.1	19.7
Crude fat (% of DM)	4.2	3.9	1.0	2.8	5.8
NDF (% of DM)	32.9	32.5	3.8	26.5	43.5
Starch (% of DM)	21.1	22.5	4.8	9.8	30.5
OM (% of DM)	92.5	92.9	1.4	90.1	94.3
Fermentable OM (% of DM)	53.2	53.3	1.1	51.0	55.0
OM digestibility (% of OM)	77.1	76.4	1.6	75.1	81.2
Roughage proportion (% of diet DM)	61.9	60.3	7.0	38.0	70.0
3-NOP dose (mg/kg DM)	70.5	61.0	25.9	37.0	137.0
CH_4_ production (g/d)	361.0	362.0	83.5	132.0	525.0
MD CH_4_ production (g/d)	−135.0	−141.0	41.9	−240.0	−64.0
Relative MD CH_4_ production (% of control)	−31.6	−30.8	10.0	−64.5	−15.1
CH_4_ yield (g/kg DMI)	16.0	17.0	3.5	7.2	23.5
MD CH_4_ yield (g/kg DMI)	−5.5	−5.4	1.7	−10.6	−2.7
Relative MD CH_4_ yield (% of control)	−29.5	−28.9	9.4	−59.6	−15.9
CH_4_ intensity (g/kg EcM)	10.6	10.5	2.8	4.3	17.1
MD CH_4_ intensity (g/kg EcM)	−4.0	−3.9	1.2	−6.2	−1.3
Relative MD CH_4_ intensity (% of control)	−31.4	−29.3	9.1	−59.1	−12.9

1 Summaries of all diets (including control) except for mean difference (MD) characteristics, where values relate to 3-nitrooxypropanol (3-NOP) treatment mean compared with control treatment mean.

**Table 2.** Estimates of overall 3-nitrooxypropanol (3-NOP) effect size and of explanatory variables from models for relative mean difference in CH_4_ production

Variable^1^	Individual model^2^			Dose + NDF			Selected^3^		
	Estimate	SE	P-value	Estimate	SE	*P*-value	Estimate	SE	*P*-value
DMI (kg/d)	−0.343	0.395	0.398						
CP (% of DM)	−0.927	0.723	0.217						
Crude fat (% of DM)	1.675	1.376	0.240				3.080	1.343	0.036
NDF (% of DM)	0.633	0.252	0.023	0.633	0.252	0.023	0.915	0.341	0.016
Starch (% of DM)	−0.073	0.207	0.728						
OM (% of DM)	0.533	1.000	0.601						
Fermentable OM (% of DM)	−1.999	1.596	0.227						
OM digestibility (% of OM)	−0.921	1.002	0.371						
Roughage proportion (% of DM)	0.011	0.242	0.965						
Overall mean	Always included			−32.8	1.6	<0.001	−32.4	1.3	<0.001
3-NOP dose^4^ (mg/kg DM)				−0.285	0.074	0.001	−0.282	0.069	0.001

1 All variables were centered to their mean value presented in Table 1.

2 Models that include an overall mean (intercept), 3-NOP dose (mg/kg DM), and a single nutritional component.

3 Selected according to smallest root mean square error from a leave-one-out cross validation.

4 Model estimate with 3-NOP dose only is −32.7 ± 1.5 − 0.313 ± 0.083 × 3-NOP (*P-*value = 0.001).

**Table 3.** Estimates of overall 3-nitrooxypropanol (3-NOP) effect size and of explanatory variables from models for relative mean difference in CH_4_ yield

Variable^1^	Individual model^2^			Dose + NDF			Selected^3^		
	Estimate	SE	*P*-value	Estimate	SE	*P*-value	Estimate	SE	*P*-value
DMI (kg/d)	−0.352	0.388	0.377						
CP (% of DM)	−0.526	0.767	0.502						
Crude fat (% of DM)	1.574	1.740	0.378				3.871	1.681	0.036
NDF (% of DM)	0.647	0.186	0.003	0.647	0.186	0.003	0.906	0.318	0.012
Starch (% of DM)	−0.226	0.225	0.328				−0.337	0.171	0.067
OM (% of DM)	0.387	1.110	0.731						
Fermentable OM (% of DM)	−1.497	1.605	0.364						
OM digestibility (% of OM)	−0.603	0.969	0.542						
Roughage proportion (% of DM)	0.135	0.231	0.568						
Overall mean	Always included			−31.0	1.5	<0.001	−30.8	1.5	<0.001
3-NOP dose^4^ (mg/kg DM)				−0.231	0.069	0.004	−0.226	0.064	0.003

1 All variables were centered to their mean value presented in Table 1.

2 Models that include an overall mean (intercept), 3-NOP dose (mg/kg DM), and a single nutritional component.

3 Selected according to smallest root mean square error from a leave-one-out cross validation.

4 Model estimate with 3-NOP dose only is −30.9 ± 1.5 − 0.267 ± 0.083 × 3-NOP (*P-*value = 0.005).

**Table 4.** Estimates of overall 3-nitrooxypropanol (3-NOP) effect size and of explanatory variables from models for relative mean difference in CH_4_ intensity

Variable^1^	Individual model^2^			Selected^3^		
	Estimate	SE	*P*-value	Estimate	SE	*P*-value
DMI (kg/d)	−0.775	0.342	0.037			
CP (% of DM)	−0.215	0.628	0.736			
Crude fat (% of DM)	0.455	1.291	0.729			
NDF (% of DM)	0.723	0.167	<0.001	0.723	0.167	<0.001
Starch (% of DM)	−0.235	0.161	0.161			
OM (% of DM)	−0.203	0.804	0.804			
Fermentable OM (% of DM)	−0.974	1.323	0.472			
OM digestibility (% of OM)	0.059	0.971	0.952			
Roughage proportion (% of DM)	0.052	0.177	0.773			
Overall mean	Always included			−33.0	1.2	<0.001
3-NOP dose^4^ (mg/kg DM)				−0.275	0.054	<0.001

1 All variables were centered to their mean value presented in Table 1.

2 Models that include an overall mean (intercept), 3-NOP dose (mg/kg DM), and a single nutritional component.

3 Selected according to smallest root mean square error from a leave-one-out cross validation.

4 Model estimate with 3-NOP dose only is −32.6 ± 1.3 − 0.324 ± 0.066 × 3-NOP (*P-*value <0.001).
